# Perceptions of the term “abstinence” among adolescents and young adults: a qualitative study on alcohol non-consumption in Switzerland

**DOI:** 10.3389/adar.2025.13969

**Published:** 2025-02-17

**Authors:** Diana Fernandes Palhares, Lorraine Chok, Joan-Carles Suris, Yara Barrense-Dias

**Affiliations:** Department of Epidemiology and Health Systems, Center for Primary Care and Public Health (Unisanté), University of Lausanne, Lausanne, Switzerland

**Keywords:** adolescents, young adults, non-drinking, alcohol consumption, abstinence

## Abstract

As part of a broader qualitative study aimed at understanding the experiences and opinions of adolescents and young adults (AYA) who do not drink alcohol in Switzerland, participants were questioned about their perceptions of the term “abstinence” in the context of alcohol non-consumption. Twelve focus groups were conducted with 63 participants (36 females, 27 males), aged between 14 years and 20 years. Participants were grouped by gender, age (based on Swiss alcohol laws), and drinking status (non-drinker or drinker). The discussions were recorded and transcribed, and thematic content analysis was used to identify and categorize key themes. The terms “abstinence,” “abstainer,” and “abstention” were generally considered unsuitable when describing young people who do not consume alcohol regardless of the drinking status of the participants. The connotation carried by the terms was mostly perceived as religious, sexual, negative and stigmatizing. “Abstinence” was considered more appropriate for adults who have stopped drinking due to alcohol-related issues. When referring to youths, terms such as “non-drinking,” “non-drinkers” or “alcohol non-consumption” were preferred, especially to better integrate non-drinking youths and positively highlight their choices in preventive and educational initiatives.

## Introduction

When considering alcohol non-consumption among young people, the terminology in the literature varies. Those who do not drink are referred to as “non-drinkers,” “alcohol abstainers,” or described as “abstaining” with terms like “abstinence from alcohol” or “alcohol abstention” also frequently used to designate adolescents and young adults (AYA) who do not consume alcohol. These terms are used interchangeably [1–7]. As part of a broader qualitative study [8] aimed at gathering the experiences and opinions of youths who do not drink alcohol, as well as the representations of non-consumption among youths who do in Switzerland, participants were questioned about their perceptions of the term “abstinence” in the context of alcohol non-consumption among young people.

## Method

Twelve focus groups were conducted between April-June 2022, each lasting approximately 1 hour, with a total of 63 participants (36 females, 27 males), all aged between 14 years and 20 years. The groups were divided by gender, age categories based on legal Swiss legal thresholds for alcohol consumption (14-15 – prohibited consumption, 16-17 - permitted consumption for beer, cider and wine, and 18–20 – permitted consumption) and drinking status (drinker or non-drinker). Due to ongoing COVID-19 health measures at our institute and the reluctance among most young people to attend in-person discussions—possibly influenced by habits formed during the pandemic—there were challenges with physical attendance. As a result, while the first group met in person, the remaining 11 groups were conducted via the secure professional version of Zoom^©^. The discussions were recorded using an unconnected Dictaphone and then transcribed verbatim, with anonymization applied to any identifiable elements (e.g., first name, school name, neighbourhood) mentioned during the discussions.

The first question of the discussion was broad, aimed at gathering their opinions on the “abstinence” in the context of alcohol non-use among AYA (“What do you think about the term “abstinence” in relation to alcohol consumption among young people? What does it mean for you?”). We used MAXQDA software (v.2022) and thematic content analysis [9, 10] to extract and identify the various themes and dimensions raised by the participants and to categorize the data into thematic categories. Quotes were translated from French into English.

## Results

When brought up in younger groups (14–15 year-olds), the term abstinence was a confusing one as most of them did not understand the meaning of the word.

“I don’t know what it (abstinence) means.” (Female, 15, drinker).

Once defined, however, younger participants felt that the word was more appropriate than older ones, understanding it as an equivalent to not drinking. The difference lay in the fact that younger participants appeared less critical or less inclined to challenge the term. Some participants within the older age groups accepted the term as they consider alcohol consumption as a norm for youth and those who choose not to drink might be tempted and perceived by some as going against what is expected of them.

“The term ‘abstinent,’ I think it’s rather good… I mean, it works because there’s a sort of not giving in to temptation.” (Male, 20, non-drinker).

However, most participants felt that this term was inappropriate and gave a negative label to non-drinkers. To them, it normalized alcohol consumption since no special term was used for alcohol drinkers, which makes “abstainers” appear as those being out of the norm.

“To me, it shouldn’t be those who don’t drink alcohol who are (called) abstainers, but those who drink who should be (called) consumers […]. I think that it (the term abstinent) somehow labels people who don't drink.” (Female, 19, non-drinker).

“Abstinence” was also linked to religion and the self-restraint often associated to priesthood.

“It makes me think especially of priests who abstain, but uh I don’t really know. It’s like abstaining from all sins. That’s why it makes me think of priests, but it doesn’t necessarily make me think immediately of alcohol.” (Female, 16, drinker).

Furthermore, “abstinence” was also understood in regard to people not drinking to honor their religious beliefs.

“I agree. The word abstinence also makes me think of religion. People who don't drink on principle […].” (Female, 20, drinker).

In another similar instance, the word “abstinence” was used in the context of sexual abstinence rather than associated to alcohol.

“It has more of a connotation about sex ‘abstinence,’ but in itself it’s actually justified” (Female, 19, drinker).

In this example, the participant finally considered that this term was appropriate in the context of alcohol consumption among adolescents, as it meant an absence of action. However, alcohol abstinence was not the immediate connection she made, showing that this term is rather unusual to use in the context of adolescents’ alcohol non-consumption.

A majority of the older participants (16–20 year-olds) reported that the term “abstinence,” or the subsequent “abstinent,” was unsuitable when talking about young people who chose not to consume alcohol. Indeed, according to them, the terminology “abstinent from alcohol” would be more fitting when talking about adults.

“*But I think we would use that term more to refer to [*…*], for example, our parents or the generation before. [*…*] (When) we talk about a person who abstains from alcohol [*…*] I would think more of an older person, like 40 years old, I don’t know. [*…*] Because he or she has developed an addiction or because of health problems [*…*].” (Female, 17, drinker).*

For them, “abstinence” from alcohol was related to specific reasons encountered in adulthood, such as alcoholism or other health problems, issues that affect or concern youth less. They suggested that the person had to quit drinking because of heavy alcohol consumption to be considered “alcohol abstainer.”

“For me, when we talk about the term ‘abstinent,’ it’s more negative or […] connected with the idea of Alcoholics Anonymous […]. The person stopped drinking completely, because it became vital to stop.” (Male, 16, non-drinker).

Furthermore, the concept of “abstinence” was often considered in association with the idea of “deprivation,” a refusal to do something – in this case drink alcohol, despite a desire to do so.

“I also think there is this notion of desire in the word ‘abstinence.’ I think that to be able to say ‘I’m abstinent,’ you have to wish to drink and refuse it.” (Female, 17, non-drinker).

For them, not drinking was above all a choice that was not experienced as restrictive.

## Discussion

The terms “abstinence,” “abstainer,” or “abstention” were frequently considered unsuitable when addressing young people who do not consume alcohol regardless of their drinking status. These terms were also often misunderstood by younger participants. The concerned parties did not always deem it as an appropriate way to designate their non-drinking status. The connotation they can carry was often perceived as negative and stigmatizing, categorizing young non-drinkers as “out of the norm” in a cultural context where alcohol consumption is expected, which makes it harder for them to socialize [11]. Furthermore, the term “abstinence” was considered as more relevant for adults who had stopped drinking due to problems such as health concerns or alcoholism, rather than for adolescents in the early stages of alcohol exposure. It is precisely in this sense that some articles have used “abstinence” to examine its effectiveness in alcohol use disorder treatment [12–15].

Rather than fostering open communication, these terms may create a divide between young people and prevention messages, while they have precisely called for increased visibility of non-drinkers in prevention efforts to raise awareness among drinkers about the risk of judgment and give opportunities for empowerment [8]. Continuing to use “abstinence” in literature and prevention campaigns to describe young non-drinkers may therefore be detrimental, further marginalizing a group that already feels outside the norm [16, 17], and discouraging them from engaging in conversations about health and risky behaviors.

Similar recommendations emerged from a qualitative study [18] exploring the experiences of undergraduate students at the University of Nottingham who drink little or no alcohol. Findings showed that non-drinkers often feel compelled to justify or conceal their choices to avoid stigmatization within a university culture where drinking is perceived as a norm. The study highlighted the importance of fostering better understanding and dialogue between drinkers and non-drinkers to reduce social exclusion and challenge stereotypes. Using stigmatizing terms like “abstinent” is unlikely to aid this effort, as it may reinforce negative connotations associated with non-drinking and deepen the divide between these groups. Instead, adopting more neutral and inclusive language can promote mutual respect, support and inclusivity.

Banister et al. [19] provide a deeper sociological analysis of non-drinkers by conceptualizing them as individuals defined by what they do not do. The authors argue that the “reverse marking” of non-drinking, where the absence of alcohol consumption is noted and observed, generates significant negative connotations. This process demonstrates how the cultural norm of drinking frames non-drinkers as “other.” In relation to the term “abstinent,” the findings of Banister et al. underscore the risk of framing non-drinkers with labels that emphasize absence or deviation from the norm. Terms like “abstinent” may reinforce the “othering” process identified in their study, embedding the negative connotations associated with non-drinking further.

### Strengths and limitations

This study has the ability to provide nuanced insights into young people’s perceptions and the potential to inform culturally relevant communication strategies aimed at promoting alcohol non-consumption in a non-stigmatizing manner. While the results may not be generalizable to a larger population, they provide valuable insights into individual experiences and the meanings attributed to the term “abstinence” among young people. Researcher bias, which can influence the interpretation of data, is inherent in qualitative research. However, this bias was mitigated with triangulation by involving several researchers in the analysis, reducing the impact of personal biases and increasing the validity of the findings. Qualitative research relies on subjective data, such as participants’ opinions and perceptions, which may lead to varied interpretations. That said, subjectivity is at the core of qualitative research, which aims to understand phenomena in context and explore the diversity of viewpoints, and the complexity and nuances of human experiences. The findings of this study may be specific to the cultural or social context in which it was conducted, especially in a setting like Switzerland, which is characterized by a strong wine culture. This context-specific focus could provide valuable insights that can inform localized interventions or policies, while offering a foundation for further research in different cultural or geographic settings. Finally, one limitation of this study may stem from the fact that some discussions were conducted in person while others took place online. This difference in format could potentially influence participants’ comfort levels, particularly in the online setting. However, every effort was made to ensure that the online environment was as secure and welcoming as possible. It is worth noting that participants appeared to navigate the online format with ease, likely due to their familiarity with remote schooling and their adaptability to digital platforms.

## Conclusion

Adopting terms such as “non-drinker” is a significant improvement over stigmatizing labels like “abstinent” or “abstainer,” as it avoids negative connotations and establishes a more equal linguistic footing with drinkers. However, framing non-drinking purely as the absence of an action—emphasizing what individuals do not do—still reflects a cultural bias that positions drinking as the norm. This approach inadvertently reinforces the perception of non-drinking as a deviation rather than an active and valid choice.

Future reflections should explore ways to reframe non-drinking as a positive, empowered decision rather than a “non-act” to focus on the benefits of non-drinking, challenge the cultural dominance of alcohol consumption and foster greater inclusivity. By redefining non-drinking as a proactive and legitimate identity, prevention efforts can create a more supportive environment. Moving away from divisive labels and promoting greater mutual understanding between drinkers and non-drinkers is essential to foster inclusivity and reduce the cultural marking of non-drinking as an act of deviance.

## Data Availability

The datasets presented in this study can be found in online repositories. The names of the repository/repositories and accession number(s) can be found below: https://doi.org/10.16909/dataset/37.
